# P-I Snake Venom Metalloproteinase Is Able to Activate the Complement System by Direct Cleavage of Central Components of the Cascade

**DOI:** 10.1371/journal.pntd.0002519

**Published:** 2013-10-31

**Authors:** Giselle Pidde-Queiroz, Fábio Carlos Magnoli, Fernanda C. V. Portaro, Solange M. T. Serrano, Aline Soriano Lopes, Adriana Franco Paes Leme, Carmen W. van den Berg, Denise V. Tambourgi

**Affiliations:** 1 Laboratório de Imunoquímica, Instituto Butantan, São Paulo, Brazil; 2 Laboratório de Toxinologia Aplicada, Instituto Butantan, São Paulo, Brazil; 3 Laboratório Nacional de Biociências, Centro Nacional de Pesquisa em Energia e Materiais, Campinas, São Paulo, Brazil; 4 Institute of Molecular and Experimental Medicine, School of Medicine, Cardiff University, Cardiff, United Kingdom; Institut de Recherche pour le Développement, Benin

## Abstract

**Background:**

Snake Venom Metalloproteinases (SVMPs) are amongst the key enzymes that contribute to the high toxicity of snake venom. We have recently shown that snake venoms from the *Bothrops* genus activate the Complement system (C) by promoting direct cleavage of C-components and generating anaphylatoxins, thereby contributing to the pathology and spread of the venom. The aim of the present study was to isolate and characterize the C-activating protease from *Bothrops pirajai* venom.

**Results:**

Using two gel-filtration chromatography steps, a metalloproteinase of 23 kDa that activates Complement was isolated from *Bothrops pirajai* venom. The mass spectrometric identification of this protein, named here as C-SVMP, revealed peptides that matched sequences from the P-I class of SVMPs. C-SVMP activated the alternative, classical and lectin C-pathways by cleaving the α-chain of C3, C4 and C5, thereby generating anaphylatoxins C3a, C4a and C5a. *In vivo*, C-SVMP induced consumption of murine complement components, most likely by activation of the pathways and/or by direct cleavage of C3, leading to a reduction of serum lytic activity.

**Conclusion:**

We show here that a P-I metalloproteinase from *Bothrops pirajai* snake venom activated the Complement system by direct cleavage of the central C-components, *i.e.*, C3, C4 and C5, thereby generating biologically active fragments, such as anaphylatoxins, and by cleaving the C1-Inhibitor, which may affect Complement activation control. These results suggest that direct complement activation by SVMPs may play a role in the progression of symptoms that follow envenomation.

## Author Summary

The genus *Bothrops* inflicts the vast majority of snakebites in Central and South America and is responsible for 90% of snake envenomations in Brazil. Envenomations are characterized by prominent local effects, including edema and necrosis, and by systemic manifestations such as hemorrhage, coagulopathy and acute renal failure. Several components have been isolated from *Bothrops* venoms, and the snake venom metalloproteinases (SVMPs) are key enzymes contributing to the high toxicity of the venoms. Previously, we analyzed the pro-inflammatory properties of snake venoms from the genus *Bothrops* and demonstrated that several of them were potent activators of the Complement (C) system. C3a, C4a and C5a were generated in venom-treated sera not only through C-activation but also by direct cleavage of C-components. In the present study, we have isolated and characterized a metalloproteinase from *Bothrops pirajai* snake venom, named here as C-SVMP, which interferes with all three complement pathways, generating potent pro-inflammatory fragments, such as C3a, C4a and C5a. Our data suggest that C-activation by *Bothrops pirajai* venom is due to activity of an SVMP, which may play a role in the progression of symptoms that follow envenomation.

## Introduction

The venom of *Viperidae* snakes is a bioactive mixture of proteases, peptides, and non-enzymatic proteins that interact with multiple components of the body affecting the hemostatic system. The genus *Bothrops* inflicts the vast majority of snakebites in Central and South America and is responsible for 90% of snake envenomations in Brazil [1]–[3].

Envenomations are characterized by prominent local effects, including edema, hemorrhage and necrosis, which can lead to permanent disability. Systemic manifestations, such as hemorrhage, coagulopathy, shock and acute renal failure, may also occur [1], [4], [5]. Several components have been isolated from *Bothrops* venoms, including proteases such as serineproteinases and metalloproteinases, phospholipase A_2_, L-amino acid oxidase, 5′-nucleotidase, hyaluronidase and C-type lectins [6], and these comprise more than 90% of their dry weight [7]. Transcriptomic analysis showed that 30% and 8% of the total transcripts present in the venom gland of adult *Bothrops jararaca* encode metallo- and serine-proteinases, respectively [8]. These enzymes have diversified amino acid sequences and display a variety of physiological activities related to the pathogenesis of local and systemic reactions [6], [9], [10].

The snake venom metalloproteinases (SVMPs) include enzymes responsible for the cleavage of important tissue proteins such as laminin, nidogen, fibronectin, collagen type IV, and proteoglycans present in the endothelial basal membrane [9]. SVMPs, together with ADAM (A disintegrin and metalloproteinase) enzymes, are included in the M12B subfamily of zinc-dependent metalloproteinases. SVMPs are multi-domain proteins that have been stratified into three classes based on their domain composition: the mature proteins of class P-I contain only a catalytic domain; the other classes (P-II and P-III) contain additional non-catalytic domains, such as disintegrin (or disintegrin-like), cysteine-rich and C-type lectin-like domains [9].

The Complement (C) system is an integral part of the immune system, protecting the host organism against invasion and proliferation of various microorganisms. It is also involved in the removal of the body's own damaged and altered cells. The Complement system consists of three activation pathways, *i.e.*, classical, alternative and mannose binding lectin (MBL) pathways, which merge at the proteolytic activation step of C3, a central component of the system. Complement activation may generate anaphylatoxins and membrane attack complex (MAC). Anaphylatoxins (C3a, C4a, and C5a) are considered the bridge between innate and adaptive immunity, and they are responsible for controlling the local pro-inflammatory response through vasodilatation as well as chemotaxis and activation of leukocytes [11]–[15]. The regulatory mechanisms of Complement are extremely balanced and are controlled by several Complement inhibitors, such as membrane-bound Complement regulators and plasma proteins such as C1-esterase inhibitor (C1-Inh), which regulate activation of C1 and manose associated serine proteases (MASPs) of the classical and MBL Complement pathways, among others [11], [16]–[18].

Previously, we analyzed the pro-inflammatory properties of snake venoms from the genus *Bothrops* and demonstrated that several of them were potent activators of the classical Complement pathway [19]. This activation occurred in the absence of sensitizing antibody and was, in part, associated with cleavage of the C1-Inhibitor by metalloproteinases present in this venom, resulting in disruption of Complement activation control. Some of the *Bothrops* venom also activated the alternative and lectin pathways. C3a, C4a and C5a were generated in sera treated with the venom, not only through C-activation but also by direct cleavage of Complement components [19].

The Complement system plays an important role in the defense system, and also contributes to the amplification of inflammation if activated in excess or inappropriately controlled. Thus, the aim of the present study was to isolate and characterize a C-activating protease from *Bothrops pirajai* venom to further understand the role of Complement activation in the pathology of envenomation by *Bothrops* snakes.

## Materials and Methods

### Ethics statement

Human blood was obtained from healthy donors who knew the objectives of the study and signed the corresponding informed consent form approved by the ethics committee (SISNEP 03689012.3.0000.5467).

All procedures involving animals were carried out in accordance with ethical principles in animal research adopted by the Brazilian Society of Animal Science and the National Brazilian Legislation no. 11.794/08. The protocol was approved by the Institutional Animal Care and Use Committee from the Butantan Institute (permission no. 1109/13).

### Chemicals, reagents and buffers

Bovine serum albumin (BSA), ortho-phenylenediamine (OPD), 1,10 phenanthroline (Phen), ethylene diamine tetraacetic acid (EDTA), ethylene glycol bis-(β-aminoethyl ether)-N,N,N,N′-tetracetic acid (EGTA), mannan, lipopolysaccharide (LPS) and human IgM antibodies were purchased from Sigma (St. Louis, MI, USA). Tween 20 and Phenylmethylsulfonyl fluoride (PMSF) were purchased from Labsynth (Diadema, SP, Brazil) and Boehringer Ingelheim (Ridgefield, CT, USA), respectively. Rabbit anti-goat (RAG) and goat anti-rabbit (GAR) IgG labeled with horseradish peroxidase (IgG-HRPO) were from Promega Corp. (Madison, WI, USA). Rabbit IgG against C3 was from Santa Cruz Biotechnology, Inc. (Santa Cruz, CA, USA). Purified human C3, C4, C5, C1-Inh and goat anti-human C4 IgG were obtained from the Quidell Corporation (San Diego, CA, USA). Fluorescent resonance energy transfer (FRET) substrate, Abz-F-R-S-S-R-Q-EDDnp (Abz-FRSSRQ-EDDnp), was synthesized and purified according to Araújo et al. [20]. Cobra venom factor (CVF) was purified in house from *Naja naja* venom as previously described [21]. Mouse serum against rabbit erythrocytes was prepared in house. The following buffers were used: Veronal-Buffered Saline (VBS^++^), pH 7.4 (10 mM Na Barbitone, 0.15 mM CaCl_2_ and 0.5 mM MgCl_2_); BVB^++^ (VBS^++^, pH 7.2, containing 0.1% BSA); alternative pathway (AP) buffer, pH 7.4 (5 mM Na-barbital, 10 mM EGTA, 7 mM MgCl_2_ and 150 mM NaCl); BAP (AP buffer, pH 7.4, containing 0.1% BSA); BAP/Tween (BAP with 0.05% Tween 20); HEPES-buffered saline (HBS^++^), pH 7.4 (10 mM HEPES, 140 mM NaCl, 5 mM KCl, 1 mM CaCl_2_ and 1 mM MgCl_2_); phosphate-buffered saline (PBS), pH 7.2 (10 mM Na Phosphate, 150 mM NaCl); and fluorescence activated cell sorter (FACS) buffer (PBS, 1% BSA and 0·01% sodium azide).

### Venoms

*Bothrops pirajai* and *Naja naja* venoms were supplied by the Herpetology Laboratory from the Butantan Institute, São Paulo, Brazil. Stock solutions were prepared in PBS buffer at 1.0 mg/mL. The permission to access the venom of *Bothrops pirajai* (permission no. 01/2009) was provided by the Brazilian Institute of Environment and Renewable Natural Resources (IBAMA), an enforcement agency of the Brazilian Ministry of the Environment.

### Protein purification

*Bothrops pirajai* crude venom (50 mg) was applied to a FPLC-GP-250 Plus system using a molecular exclusion column (Superose 12 10/300 GL, GE Life Sciences, USA), equilibrated with 0.05 M ammonium bicarbonate, pH 7.8. Elution was carried out using the same buffer at a flow rate of 30.0 mL/h. The active fraction (screened for the ability to cleave C3) was chromatographed on a Superdex 75 10/300 GL column (GE Life Sciences, USA), using the same buffer and flow rate. Both fractionations were monitored by ultraviolet absorption (280 nm). The molecular mass and homogeneity of the active fraction was assayed (w/v) by SDS–PAGE using a 12% polyacrylamide gel [22] and silver staining [23].

### Mass spectrometry analyses

The protein digestion procedure was followed as proposed by Kinter and Sherman [24] using 20 µg of the purified protein. In the final procedure, the samples were dried using a speed-vac (CHRIST, model RVC2-18, Osterode, Germany) and dissolved in 15 µL of formic acid 0.1% (v/v). An aliquot (4.5 µL) of the resulting peptide mixture was separated by C18 column (75 µm i.d.×100 mm) (Waters, Milford, MA) on an UPLC-ESI-Q-TOF system (Waters, Milford, MA, USA) at a flow rate of 600 nL/min. The gradient was 3–45% of solvent B (0.1% formic acid in acetonitrile), 45–80% B in 2.5 min, hold at 80% B for 1 min, then back to 97% of solvent A (0.1% formic acid in deionized water) in 1.5 min. The MS instrument was operated in data dependent mode, in which one full MS scan was acquired in the *m/z* range of 200–2000 followed by MS/MS acquisition using collision-induced dissociation of the 3 most intense ions from the MS scan. The resulting fragment spectra were searched using the MASCOT search engine (Matrix Science, UK) against the randomized serpentes database (33870 sequences; 7677654 residues, downloaded from UniProt), with parent and fragment tolerances of 0.1 Da. Iodoacetamide derivatives of cysteine and methionine oxidation were specified in MASCOT as variable modifications. Only peptides that showed a significant threshold (p<0.05) in MASCOT-based score were considered.

For the determination of molecular mass, the purified toxin (200 ng) was acidified with formic acid 0.1% and analyzed by a Q-ToF Ultima API spectrometer (Waters Corp.), operated in MS continuum mode. Data were acquired from *m*/*z* 100–3.000 at a scan rate of 1 s and an interscan delay of 0.1 s. The spectra were accumulated over 30 scans, and the multiple charged data produced on the m/z scale were converted to the mass scale using the maximum entropy-based software [25] supplied with the MassLynx 4.1 software package. The processing parameters were: an output mass range of 22,000–24,000 Da at a resolution of 0.1 Da/channel; the simulated isotope pattern model was used with the spectrum blur width parameter set to 0.2 Da; and the minimum intensity ratios between successive peaks were 20% (left and right).

### Enzymatic activity

Samples of purified venom protein (0.5 µg) were mixed with 5 µM Fluorescent Resonance Energy Transfer substrate and the peptide Abz-FRSSRQ-EDDnp, in the presence or absence of 15 mM phenylmethylsulfonyl fluoride or 15 mM 1,10 phenanthroline, which are inhibitors of serineproteinases and metalloproteinases, respectively. Reactions were carried out at 37°C using a fluorescence spectrophotometer (λ_em_ = 420 nm and λ_em_ = 320 nm; Victor 3™, Perkin–Elmer, MA, USA) as described by Araújo et al. [20]. All assays were performed in duplicate and the specific proteolytic activity was expressed as micromoles of cleaved substrate *per* minute *per* µg of protein (U/µg).

### Normal human serum

Human blood samples were collected without anticoagulant and allowed to clot for 2 hours at 4°C. After centrifugation, normal human serum (NHS) was collected and stored at −80°C.

### Treatment of the normal human serum

Fifty microliters of normal human serum (NHS) were incubated with 50 µL of PBS buffer containing *Bothrops pirajai* crude venom (1 µg) or purified venom protein (0.5 µg) for 30 min at 37°C. As a negative control, NHS was incubated with PBS. After treatment, the samples were tested for residual complement activity using the enzyme-linked immunosorbant assay (ELISA).

### Complement activation assays

Microtiter plates were coated with 1 µg/well of IgM (Classical Pathway), 1 µg/well of LPS (Alternative Pathway) or 10 µg/well of mannan (Lectin Pathway) overnight at 4°C. The plates were washed three times with PBS/0.05% Tween 20 and blocked with 1% BSA in PBS for 1 h at 37°C. After washing, serial dilutions of normal human serum treated with PBS, *Bothrops pirajai* crude venom or purified active protein were added. After incubation (1 h at 37°C), the plates were washed with BVB^+2^/Tween (Classical and Lectin Pathways) or BAP/Tween (Alternative Pathway) buffers and incubated with anti-human C4 (1∶2.000; Classical and Lectin Pathways) or anti-human C3 (1∶5.000; Alternative Pathway) for 1 h at 37°C. The plates were washed three times with BVB^+2^/Tween or BAP/Tween and incubated for 1 h with the specific anti-IgG antibody conjugated with HRPO. The plates were washed and the reactions developed by adding OPD substrate according to conditions established by the manufacturers (Sigma). The absorbance was measured by an ELISA reader (Multiskan spectrophotometer EX, Labsystems, Finland) at λ492 nm. Normal human serum arbitrarily set at 1000 aU/mL was used to generate a calibration curve.

### Direct cleavage of C-components

The proteolytic activity of *B. pirajai* crude venom or the purified protease on C-components was assessed by SDS-PAGE under reducing conditions. Briefly, 3 µg of each human purified C-components, C3, C4, C5 or the C-regulatory protein C1-Inh, were incubated with crude venom (1 µg), purified toxin (0.5 µg) or PBS for 30 min at 37°C in the presence or absence of 15 mM 1,10-phenanthroline. For the kinetic assays, increasing concentrations of the purified toxin (0.01 µg–0.32 µg) were used. The cleavage of the C-components was assessed using 10% (w/v) polyacrylamide gels and silver staining. Using ImageJ 1.45 software (National Institutes of Health, Bethesda, MD), the protein band density was measured. The amount of protein under control conditions was assigned a relative value of 100%. Alternatively, for amino-terminal sequence analysis of the C3 protein fragments, gels were subsequently electrotransferred to polyvinylidene difluoride (PVDF) membranes and bands of interest were submitted to Edman degradation using the PPSQ-10 protein sequencer (Shimadzu, Tokyo, Japan).

### Detection of anaphylatoxins

Samples of NHS (50 µL) or the human purified C-components C3, C4 or C5 (3 µg) were incubated with crude venom (1 µg), purified toxin (0.5 µg) or PBS for 30 min at 37°C, and C3a/C3a desArg, C4a/C4a desArg and C5a/C5a desArg concentrations were measured using the respective ELISA kit (BD Biosciences PharMingen, CA, USA).

### Analysis of the C1-Inhibitor regulatory activity

The MicroVue C1-lnhibitor Enzyme Immunoassay (Quidel, San Diego, CA) was used to measure the amount of functional C1-Inhibitor protein (3 µg) after treatment with increasing amounts of purified toxin (0.5, 1, 2 µg) or PBS (control) in accordance with the conditions established by the manufacturer.

### Action of the purified toxin on the Complement system in a murine model

Male Balb/c mice, aged 2 months and weighing 18–22 g, were obtained from Central Animal Breeding from the Butantan Institute, SP, Brazil. To measure the action of purified venom protein on the complement system *in vivo*, BALB/c mice (groups of 3 animals) were injected i.p. with 10 µg of purified protein twice at an interval of 24 h. As positive and negative controls, animals received 10 µg of CVF or PBS buffer, respectively. Serum complement activity was measured before treatment and 24 h after the second injection. The results were representative of 3 independent assays.

### Hemolytic assay for measurement of mouse serum C-activity

Blood was collected from mice and allowed to clot on ice. Sera were separated and immediately assayed. Rabbit erythrocytes were sensitized by incubation with mouse anti-rabbit erythrocyte serum, washed and resuspended at 2% in VBS^++^. For each mouse serum to be tested, doubling dilutions in VBS^++^ were made in the wells of a 96-well plate (50 µL/well). Zero (VBS^++^) and 100% lysis (H_2_O) controls were included. Antibody-sensitized rabbit erythrocytes (50 µL) were added to each well and incubated for 60 min at 37°C. The absorbance of the supernatant was measured at 415 nm and the percent hemolysis calculated by standard methods [26].

### Statistical analysis

One-way ANOVA, followed by the Bonferroni *post*-test, were used to evaluate significant differences between control and experimental results. Statistical analysis was performed using GraphPad Prism software. Differences were considered statistically significant when *p* values were *p*<0.05, *p*<0.01 or *p*<0.001.

## Results

### Purification and identification of the *Bothrops* C-activating protease

**Figure 1:**
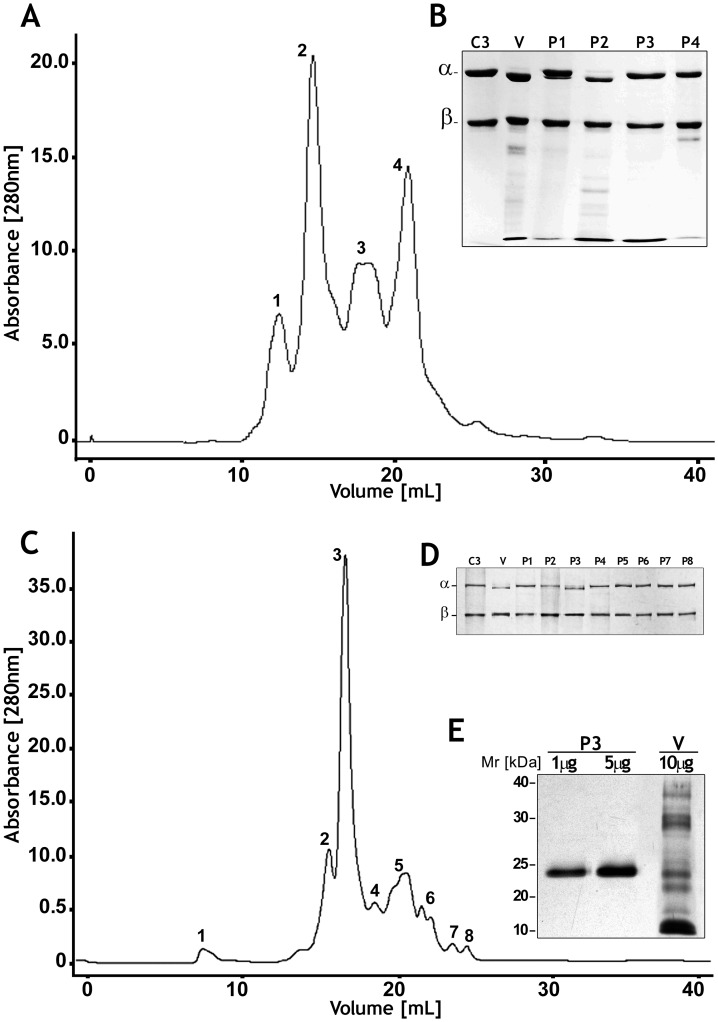
Purification of the protease responsible for Complement activation. [A] Chromatogram of *Bothrops pirajai* venom (50 mg) fractionated on a FPLC-GP-250 Plus system using a molecular exclusion column (Superose 12 10/300 GL) in 0.05 M ammonium bicarbonate buffer, pH 7.8. [B] SDS-PAGE of fractions screened by the ability to cleave component C3. [C] Chromatogram of the fraction with activity (P2) on a Superdex 75 10/300 GL column. [D] SDS-PAGE of fractions from Superdex 75 tested for the ability to cleave component C3. [E] SDS-PAGE followed by silver staining to assess the purity of P3, the fraction capable of cleaving C3.

*B. pirajai* crude venom was fractionated using a Superose 12 10/300 GL gel filtration column resulting in the elution of four chromatographic peaks (Fig. 1A). All fractions were tested for the ability to cleave purified human C3, as shown by a reduction in size of the α-chain (Fig. 1B). A subset of proteins present in the second chromatographic peak (Fig. 1A) were submitted to a second gel filtration step using a Superdex 75 10/300 GL column. The C3-cleaving activity was detected in Peak 3 (Fig. 1C, D), which showed a single protein band of 23 kDa by SDS-PAGE (Fig. 1E). Analysis by ESI-MS of intact purified protein confirmed its molecular mass as being 23145.6543 Da (Fig. S1). Analysis of the same protein by *in solution* trypsin digestion and LC-MS/MS resulted in the identification of six tryptic peptides that matched sequences of the P-I class of SVMPs from *Bothrops* venom (Table 1).

**Table 1:** Mass spectrometry analysis of the purified protein.

PROTEIN	ACCESSION NUMBER	IDENTIFIED PEPTIDES (score 191)
		DLLPR
		ERDLLPR
Snake venom metalloproteinase	P85314	YNSNLNTIR
BmooMPalfa-I		KYNSNLNTIR
		ETLKSFGEWR
		HNPQCILNEPL

The primary sequence of the protein was identified by mass spectrometry.

**Figure 2:**
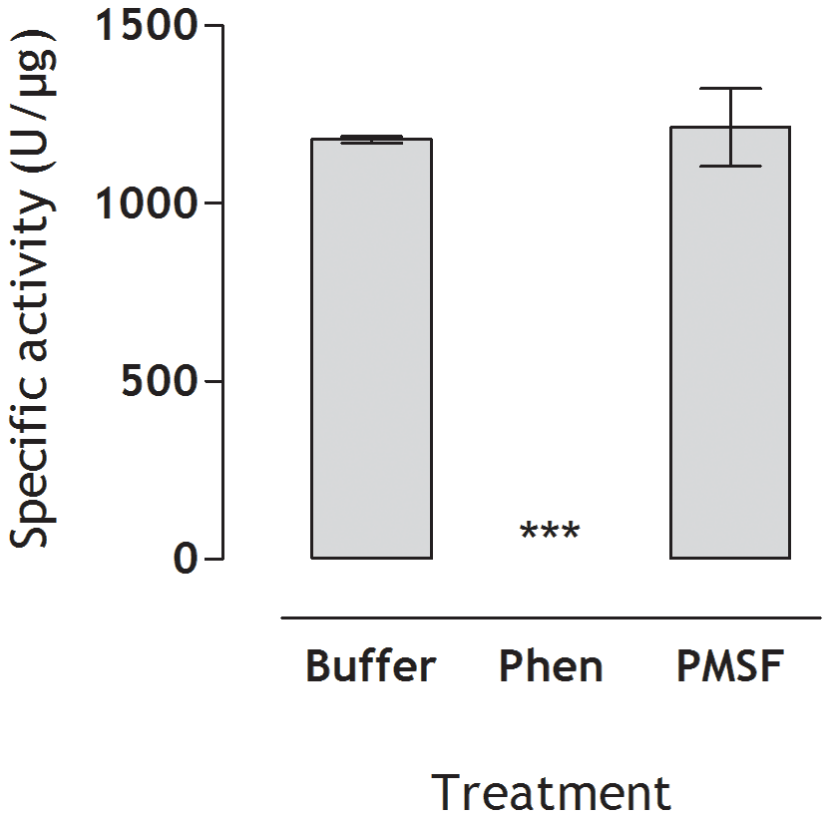
Proteolytic activity of C-SVMP upon FRET substrate. The proteolytic activity of C-SVMP (0.5 µg) on the FRET peptide (Abz-FRSSRQ-EDDnp - 5 µM in PBS) was determined in the presence or absence of 15 mM of phenylmethylsulfonyl fluoride (PMSF) or 1,10 phenanthroline (Phen). Reactions were carried out at 37°C using a fluorescence spectrophotometer (λ_em_ = 420 nm and λ_em_ = 320 nm). All assays were performed in duplicate and the specific proteolytic activity was expressed as micromoles of cleaved substrate *per* minute *per* µg of protein (U/µg).

The proteolytic activity of the purified protein, here named as C-SVMP, was tested using the FRET substrate Abz-FRSSRQ-EDDnp, which contains a universal sequence recognized by proteases of different catalytic natures. C-SVMP cleaved the FRET substrate with high activity (Fig. 2). To assess and confirm the enzymatic nature of the C-SVMP, the assay was performed in the presence of 1,10-phenanthroline or phenylmethylsulfonyl fluoride, which are inhibitors of metallo- and serine-proteinases, respectively. The proteolytic activity of C-SVMP was completely inhibited by Phen, while PMSF had no effect, identifying C-SVMP as a metalloproteinase.

### C-SVMP cleaves the α-chain of C3 in a dose dependent manner to generate biologically active C3a

**Figure 3:**
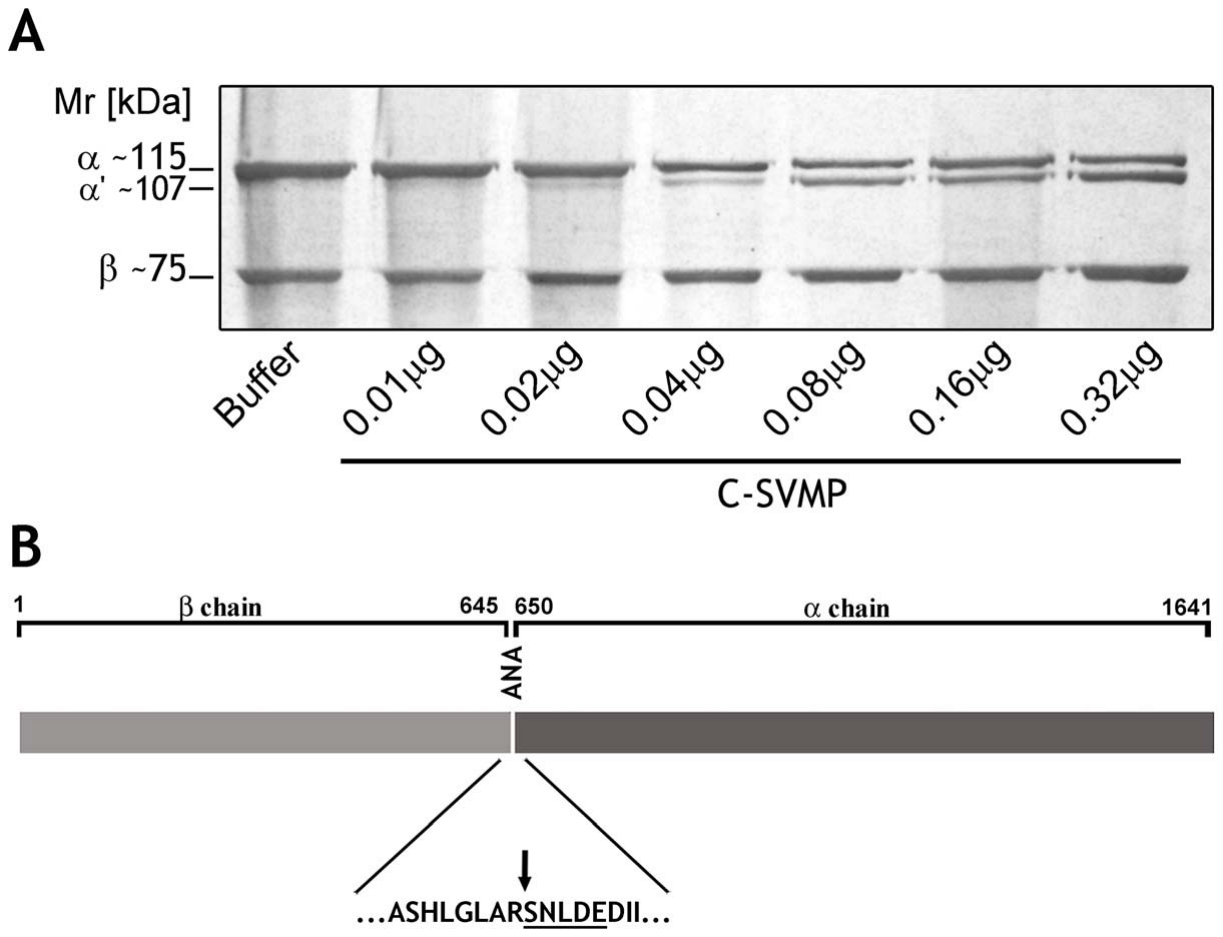
Cleavage of purified human component C3. [A] Samples of purified human C3 (3 µg) were incubated with increasing concentrations of C-SVMP (0.01–0.32 µg) or PBS for 30 min at 37°C. Cleavage was visualized by SDS-PAGE under reducing conditions followed by silver staining. [B] C3 is formed by two protein chains, β and α (1,641 amino-acid residues). The arrow indicates the site of cleavage for C-SVMP and the amino acids determined by Edman degradation are underlined. Anaphylatoxin Domain (ANA).

The action of purified C-SVMP on C3 was assessed by SDS-PAGE. C-SVMP dose-dependently hydrolyzed the alpha chain of purified human C3, resulting in a slightly lower Mr, while the beta chain was not affected (Fig. 3A). Analysis by Edman degradation of the cleaved C3 alpha chain revealed the N-terminal amino acid sequence Ser-Asn-Leu-Asp-Glu, suggesting that C-SVMP cleaves the C3 alpha chain at the same site as C3 convertases after Complement activation, thus generating C3b and C3a (Fig. 3B).

### C-SVMP interferes with the classical, alternative and lectin complement pathways

**Figure 4:**
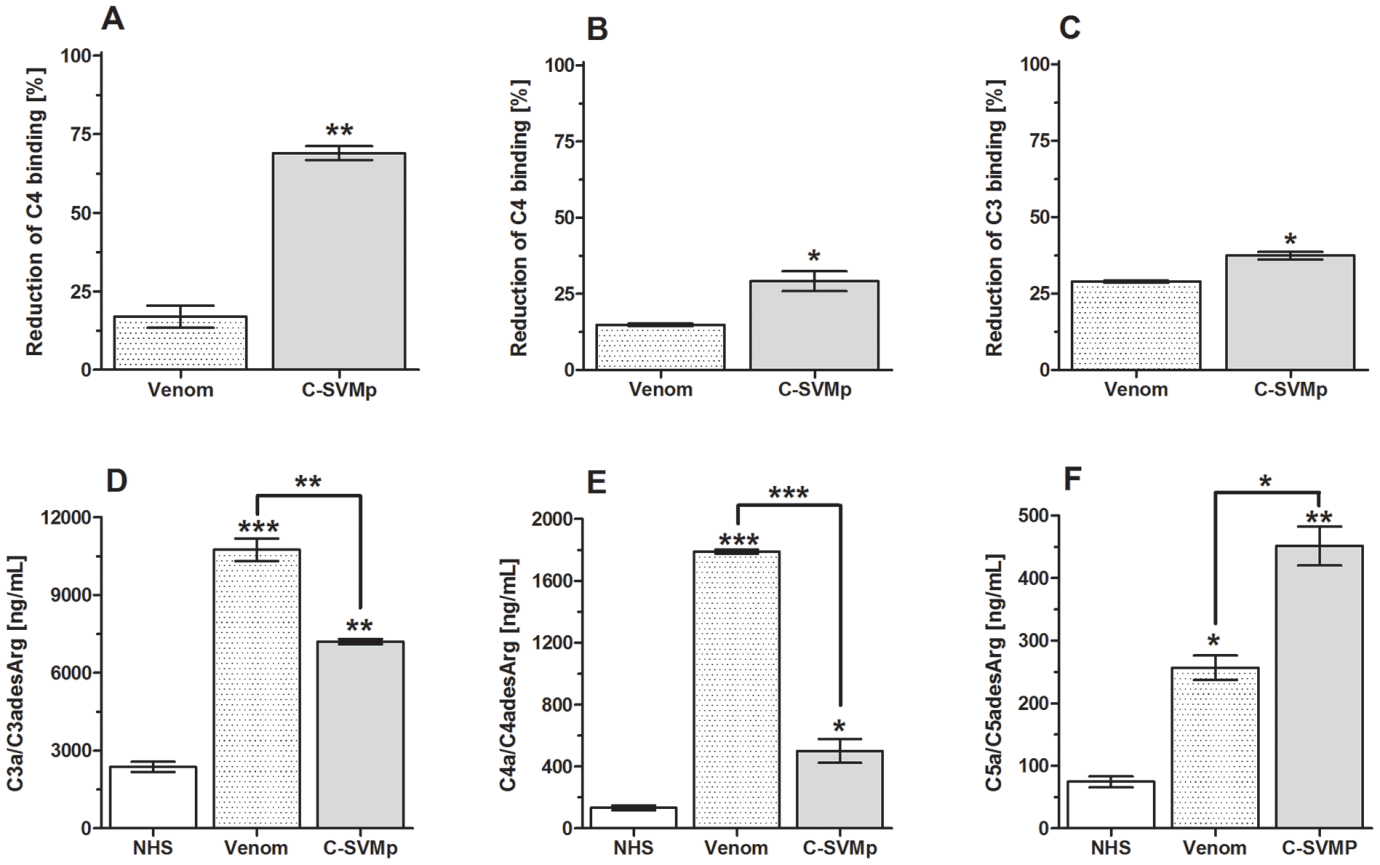
Action of C-SVMP on complement pathways. Samples (50 µl) of normal human serum (NHS), as the complement source, were incubated for 30 min at 37°C with 50 µl of C-SVMP (0.5 µg) and with 50 µl of *Bothrops* venom (1 µg) or PBS as positive and negative controls, respectively. The residual complement activity was determined by ELISA for the Classical [A], Alternative [B] and Lectin [C] pathways. The results were expressed as the percentage reduction of component deposition relative to the negative control sample (NHS+PBS). Generation of anaphylatoxins in the serum samples was measured using ELISA kits for C3a [D], C4a [E] and C5a [F]. The results were expressed as the concentration of each anaphylatoxin *per* mL of human serum. Data are representative of three separate experiments and are expressed as the mean of the duplicates +/− SD. *p<0.05; **p<0.01; ***p<0.001.

We previously showed that crude *Bothrops* spp. venoms interfered with all three Complement activation pathways [19]. To assess whether C-SVMP could also affect the Complement activation pathways, a microtiter plate-based assay was used. Human serum was pre-incubated with C-SVMP and the remaining Complement activity was measured. Similar to crude venom, C-SVMP induced a reduction in the deposition of Complement components (C4b, as determined for the classical and lectin pathways – Figs. 4 A and B; C3b for the alternative pathway - Fig. 4C), demonstrating that pre-incubation with C-SVMP resulted in Complement activation and consumption. The consumption of the classical pathway was approximately 2-fold greater than that of the alternative and lectin pathways.

Reduction in C4b and C3b deposition could also have resulted from direct inhibition, rather than from activation and consumption. The activating rather than inhibitory action of C-SVMP on the complement system cascade was confirmed by detection of three anaphylatoxins, *i.e.*, C3a, C4a and C5a (Figure 4D–E). The generation of C3a and C4a after C-SVMP treatment was approximately 3-fold higher than the untreated sera; and C5a was approximately 6-fold higher. Interestingly, C5a generation induced by C-SVMP was greater than that induced by whole venom, while C3a and C4a generation induced by C-SVMP was less.

### C-SVMP directly cleaves C4, C5 and the C1-Inhibitor

**Figure 5:**
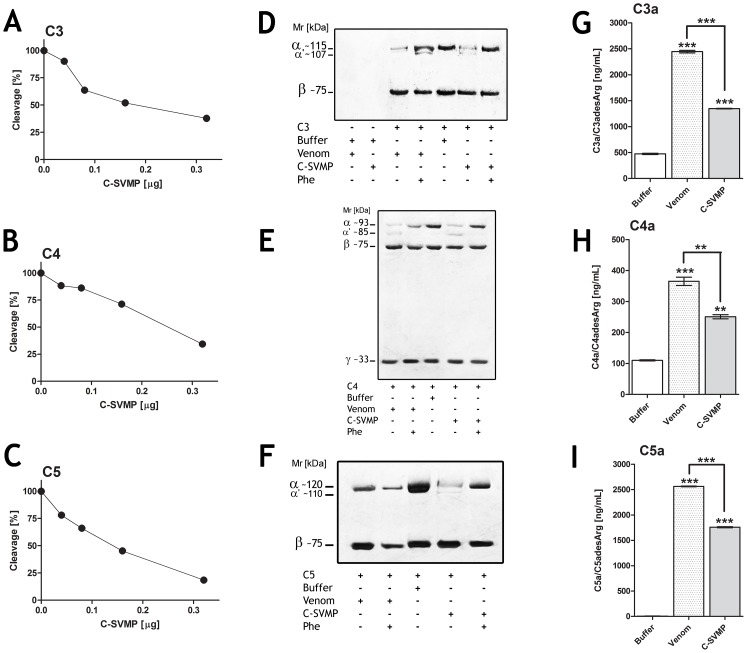
Proteolytic activity of C-SVMP on human purified Complement components. The C-components, C3, C4 and C5 (3 µg) were incubated with increasing concentrations of C-SVMP (0.01 µg–0.32 µg). Cleavage of C-components was visualized by SDS-PAGE (10%) under reducing conditions followed by silver staining. The percentage cleavage of the α chain (C3, C4 and C5) was quantified by densitometry [A–C]. Purified human complement proteins C3, C4 and C5 (3 µg) were also incubated with purified C-SVMP (0.5 µg) or *Bothrops* venom (1 µg) in the presence or absence of 1,10 phenanthroline (Phe - 15 mM), a metalloproteinase inhibitor [D–F]. Generation of anaphylatoxins, after treatment of C3, C4 or C5 samples (3 µg) with C-SVMP (0.5 µg) or *Bothrops* venom (1 µg), was determined by ELISA [G–I]. Data are representative of three separate experiments. **p<0.01; ***p<0.001.

We have previously shown that crude *Bothrops* spp. venoms directly cleaved not only C3, which was used here as a guide during the isolation of C-SVMP, but also complement components C4, C5 and the inhibitor of the classical and lectin pathways, the C1-Inhibitor [19]; therefore, the possible direct cleavage of these components by C-SVMP was assessed by SDS-PAGE and densitometry. Figure 5 (A–C) shows that C-SVMP incubation with C3, C4 and C5 resulted in a dose dependent hydrolysis of the alpha chains of these C-components, as shown by a reduced Mr of the alpha chains (Fig. 5 D–F); the Mr of the beta and gamma (C4 only) chains were not affected. Concomitantly with the reduction in Mr of the alpha chains, C3a, C4a and C5a fragments, as detected by specific ELISA, were generated (Fig. 5 G–I).

Cleavage of the alpha chains of C3, C4 and C5 by C-SVMP was completely inhibited by 1,10-phenanthroline; however, this compound only partially inhibited the action of venom on these proteins (Fig. 5 D–F), suggesting that crude venom, in addition to the metalloproteinase C-SVMP isolated here, contains another protease, most likely a serineproteinase.

**Figure 6:**
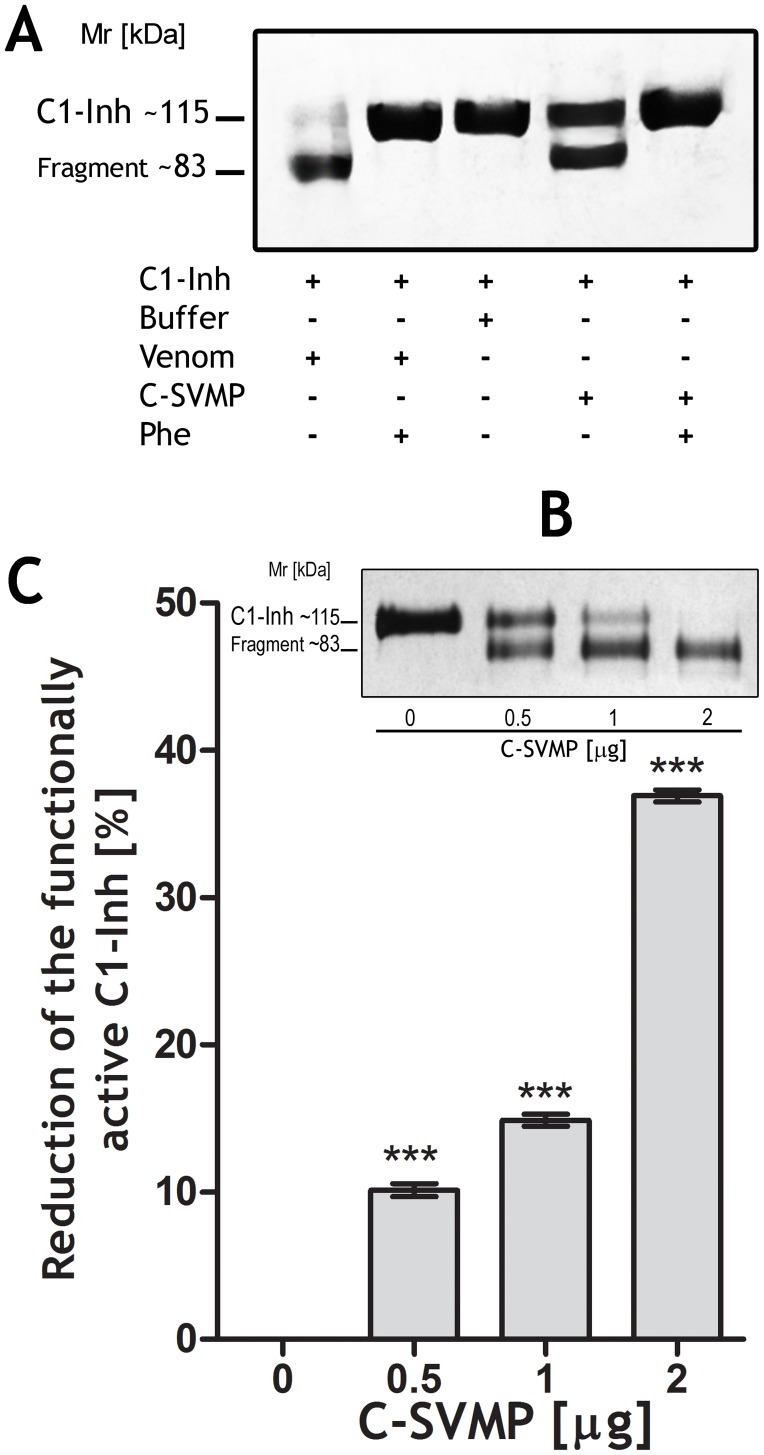
Proteolytic activity of C-SVMP on C1-Inh and analysis of its regulatory activity. [A] Human purified C1-Inh samples (3 µg) were incubated with purified C-SVMP (0.5 µg) or *Bothrops* venom (1 µg) in the presence or absence of 1,10 phenanthroline (Phe - 15 mM). [B] Alternatively, samples of purified human C1-Inh (1 µg) were incubated with increasing concentrations of C-SVMP (0.5 µg, 1 µg and 2 µg). The cleavage was visualized by SDS-PAGE (10%) under reducing conditions followed by silver staining. [C] C1-Inh activity was determined using the MicroVue ELISA kit (***p<0.001). Data are representative of two separate experiments.

The cleavage of C1-Inh was completely inhibited by 1,10-phenanthroline after treatment with either C-SVMP or crude venom (Fig. 6A), suggesting that C-SVMP may be solely responsible for cleavage of C1-Inh. Moreover, C-SVMP also dose dependently cleaved C1-Inh, generating a fragment of ∼83 kDa, which was positively associated with the dose dependent reduction in the level of the functionally active C1-Inhibitor protein (Fig. 6B,C).

### C-SVMP is also able to activate the complement system *in vivo*

**Figure 7:**
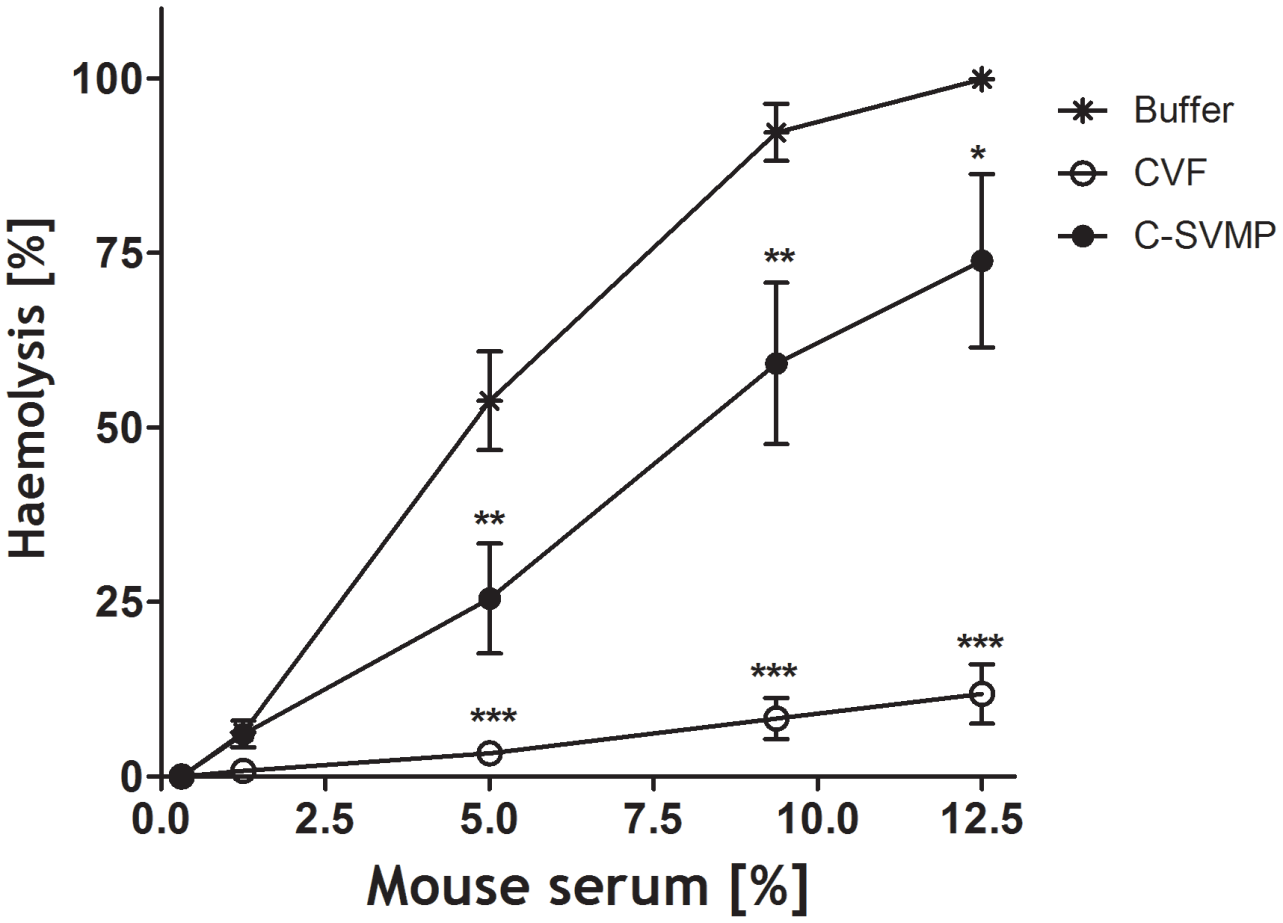
The complement activation property of C-SVMP measured *in vivo*. BALB/c mice (n = 3) were twice injected i.p. with C-SVMP (10 µg/dose) at intervals of 24 h. As positive and negative controls, animals received CVF (10 µg/dose) or PBS, respectively. Serum samples were collected immediately prior to starting treatment and at 24 h after the last injection. Complement activity in the individual serum samples was measured and expressed as the percentage of hemolysis. The results were representative of three independent assays. *p<0.05; **p<0.01; ***p<0.001.

Because C-SVMP is able to activate human complement, as determined in *in vitro* assays, we analyzed its action *in vivo* using a murine model. As a positive control, mice were treated with CVF, a well-known complement activating molecule isolated from the venom of *Naja naja*. Figure 7 shows that C-SVMP induced partial depletion of the complement system *in vivo*, as determined by the significantly reduced hemolytic activity in sera from treated mice compared to the sera of untreated animals. Under the conditions used, C-SVMP was less effective in consuming complement than CVF.

## Discussion

Complement activation by an animal's toxin and its role in the envenomation remains poorly studied, but has been described for some snake venoms belonging to the *Elapidae* and *Viperidae* families [27]–[33], [19].

The best-described complement activating protein isolated from snake venom is CVF, a C3-like molecule that complexes with factor B, which is cleaved and activated by factor D, generating a very stable C3/C5-convertase that leads to excessive complement activation and depletion [27]. There is no evidence that CVF has enzymatic activity upon individual components of the complement system, and it can only indirectly trigger the activation of the system.

In this study we have isolated and characterized a metalloproteinase from *Bothrops* snake venom, C-SVMP, that interferes with the complement system. SVMPs are known for their key role in *Bothrops* snake envenomation, producing prominent local tissue damage characterized by edema, pain, hemorrhage, dermonecrosis and inflammatory reaction, as well as by systemic effects, such as coagulopathies, nephrotoxicity, hemodynamic dysfunction and cardiotoxicity [34]–[37].

C-SVMP has a single polypeptide chain with a molecular mass of 23.145 kDa (Fig. 1 and S1) and exhibits high homology with P-I class zinc metalloproteinases from *Bothrops* venom (Table 1), *i.e.*, BmooMPalfa-I from *B. moojeni* (accession number: P85314) [38], based on a partial sequence obtained by trypsin digestion and mass spectrometry. Recently, it was isolated from the *B. pirajai* venom a new P1-class metalloproteinase with fibrin(ogen)olytic and thrombolytic activities [39], whose identity with the C-SVMP still needs further analysis, once the internal sequences obtained are similar to others P-I SVMP from *Bothops* spp. Despite the high similarity between those SVMP, they might act in specific substrates.

The metalloproteinase activity of C-SVMP was confirmed by cleavage of the FRET substrate peptide Abz-FRSSRQ-EDDnp (Fig. 2), which was inhibited by 1,10-phenanthroline but not by phenylmethylsulfonyl fluoride, thereby confirming that C-SVMP is a metalloproteinase.

Members of the P-I class of SVMPs, the small SVMPs, have molecular masses of 20–25 kDa. The catalytic domain contains a zinc-binding sequence, HEXXHXXGXXH, plus a Met-Turn sequence, CI/VM, which stabilizes the three Histidine residues important for enzymatic catalysis of the substrates; the zinc binding region is strictly conserved among the SVMPs [9].

Numerous biological activities are attributed to P-I class SVMPs in the context of the envenoming pathology, such as fibrinolysis and fibrinogenolysis, hemorrhage, myonecrosis, inflammation, apoptosis and prothrombin activation in addition to inhibition of platelet aggregation activities [34]–[39]. However, the effect on the complement system has not been investigated.

Our current data show that C-SVMP, isolated based on its C3-cleaving ability, interferes with all three complement pathways but preferentially interferes with the classical pathway. C-SVMP generated bioactive fragments, such as C3a, C4a and C5a, as confirmed by ELISA (Fig. 4), and C3b, as determined by the amino acid sequence analysis of the cleaved N-terminal C3-alpha chain, Ser-Asn-Leu-Asp-Glu (Fig. 3). This cleavage site is identical to that accomplished following complement activation via any of the three activation pathways [40].

Interestingly, Sun and Bao [41] have isolated from *Naja atra* venom a PIII metalloproteinase (termed atrase B) with anticomplementary activity. This protease shows fibrinogenolytic activity and edema-inducing activity, but has no hemorrhagic activity. Atrase B inhibits the hemolytic activity of the complement system by cleaving C-components C6, C7, C8 and factor B, thus affecting MAC assembly. Moreover, differently from C-SVMP from *B. pirajai*, atrase B has no enzymatic activity on C1, C2, C3, C4 and C5.

In addition to cleaving and activating C3, C4 and C5 (Figs. 4 and 5), C-SVMP also directly cleaved C1-Inh (Fig. 6), a regulatory protein that binds and inactivates C1r, C1s, MASP-1 and MASP-2, as well as interfering with the regulation of the coagulation cascade. C1r and C1s activate the classical complement pathway, while MASP-1 and MASP-2 activate the lectin pathway [42]. Cleavage of C1-INH by Crotalid, Viperid and Colubrid snake venoms has previously been reported by Kress et al. [43], who demonstrated that the inhibitor was converted into an active 89-kDa intermediate species and then further cleaved to form an 86-kDa inactive inhibitor. Inactivation, even partial, of the C1-Inhibitor by C-SVMP action (Fig. 6) may lead to auto-activation of the classical and lectin pathways [18], [44]. The more potent action of C-SVMP on the classical pathway compared to the lectin pathway may possibly be explained by stronger inhibition of the classical pathway by C1-Inh, although this remains to be investigated. Inactivation of C1-Inh not only results in uncontrolled C-derived anaphylatoxin generation but also uncontrolled bradykinin generation [18], [44]; all of these products contribute to vasodilatation and increased spreading of the venom.

*In vivo*, C-SVMP induced consumption of murine complement components most likely via activation of the classical pathway and/or direct cleavage of C3, thereby reducing lytic activity of the sera (Fig. 7). The reduction of serum lytic activity was not as great as the reduction observed following injection of CVF, showing that CVF is more efficient in dysregulating the complement system. This may be explained by the different dysregulation mechanisms of the complement system; while C-SVMP causes dysregulation by direct enzymatic cleavage, CVF forms a very stable C3/C5 convertase with factor B, which has an *in vivo* half-life of 11.5 h [45] and thus can cause prolonged C-activation. Furthermore, due to its smaller size (Mw 23 kDa), C-SVMP may get cleared from circulation more easily than the much larger CVF (Mw 150 kDa, which increases to over 200 kDa when complexed with fB, thereby extending the *in vivo* half-life).

In conclusion, we show here that a P-I metalloproteinase from *Bothrops pirajai* snake venom is able to activate the complement system by direct cleavage of central C-components, *i.e.*, C3, C4 and C5, thereby generating biologically active fragments, such as anaphylatoxins, and by cleaving the C1-Inhibitor, which may affect Complement activation control. These results suggest that direct complement activation by SVMPs may play a role in the progression of symptoms following envenomation.

## Supporting Information

Figure S1 Molecular mass determination of C-SVMP (300 ng) was performed by electrospray ionization (ESI) coupled with mass spectrometry using a Q-ToF instrument.
(TIF)
