# Supplementary figures and images for: P-I Snake Venom Metalloproteinase Is Able to Activate the Complement System by Direct Cleavage of Central Components of the Cascade
Source: PLoS Negl Trop Dis. 2013 Oct 31;7(10):e2519. doi: 10.1371/journal.pntd.0002519 (PMC3814341; doi:10.1371/journal.pntd.0002519)

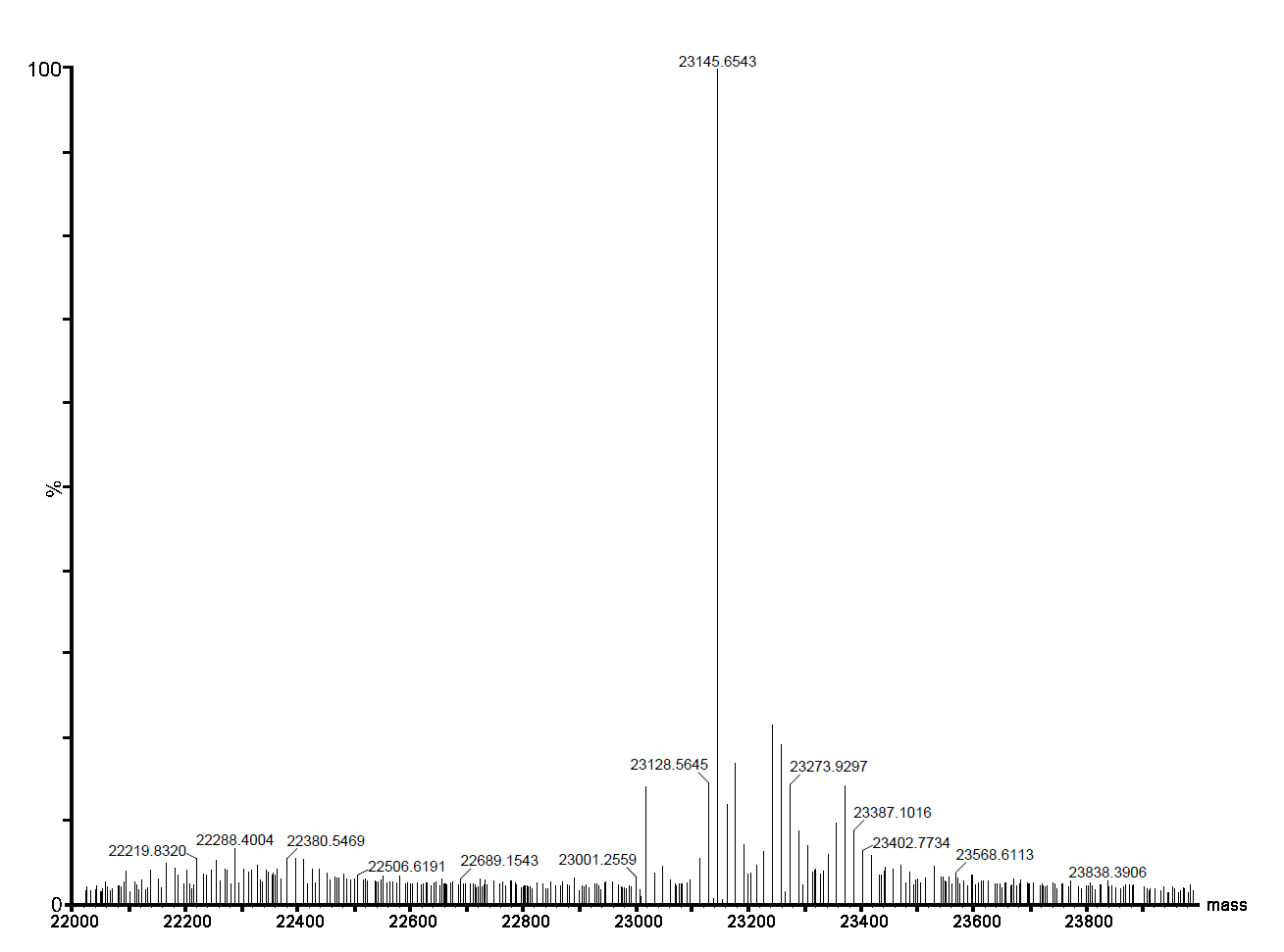

Supplement: Figure S1 — Molecular mass determination of C-SVMP (300 ng) was performed by electrospray ionization (ESI) coupled with mass spectrometry using a Q-ToF instrument. (TIF) [file pntd.0002519.s001.tif]
